# Retraction: Lipoma Growing on the Back for 26 Years: A Bizarre Case Report

**DOI:** 10.7759/cureus.r142

**Published:** 2024-06-17

**Authors:** Samiksha V Gupta, Shubham Durge, Nachiket P Rahate, Prashant V Rahate

**Affiliations:** 1 Psychiatry and Behavioral Sciences, Jawaharlal Nehru Medical College, Datta Meghe Institute of Higher Education and Research, Wardha, IND; 2 General Surgery, Jawaharlal Nehru Medical College, Datta Meghe Institute of Higher Education and Research, Wardha, IND; 3 Surgery, Seven Star Hospital, Nagpur, IND

This article has been retracted by the Editor-in-Chief after it has been discovered that this case was already presented and published in Kher C, Chakole S (January 26, 2024) Giant Lipoma: A Case Report. Cureus 16(1): e53000. doi:10.7759/cureus.53000.

